# The synergistic antitumor effect of arsenic trioxide combined with cytotoxic T cells in pulmonary metastasis model of colon cancer

**DOI:** 10.18632/oncotarget.22757

**Published:** 2017-11-30

**Authors:** Lei Wang, Wentao Liang, Na Peng, Xiang Hu, Yingxin Xu, Zhong Liu

**Affiliations:** ^1^ Department of General Surgery, First Affiliated Hospital of Dalian Medical University, Dalian 116011, China; ^2^ The United Innovation of Mengchao Hepatobiliary Technology Key Laboratory of Fujian Province, Mengchao Hepatobiliary Hospital of Fujian Medical University, Fuzhou 350025, China; ^3^ Institute of General Surgery, Chinese PLA General Hospital, Beijing 100853, China; ^4^ Zhongnan Hospital of Wuhan University, Institute of Hepatobiliary Disease of Wuhan University, Transplant Center of Wuhan University, Hubei Key Laboratory of Medical Technology on Transplantation, Wuhan 430071, China

**Keywords:** arsenic trioxide, cytotoxic T cells, regulatory T cell, colon cancer, adoptive T cell therapy

## Abstract

Adoptive T cell therapy, including cytotoxic T lymphocytes (CTLs), represents a promising non-toxic anticancer strategy. The effects of this therapy can be impaired by tumor-infiltrated regulatory T cells (Tregs). Autologous murine CTLs acquired using cryopreservation exhibited a cytotoxic effect equivalent to that of conventional CTLs. The killing activity of CTLs was enhanced significantly using arsenic trioxide (ATO), accompanied by reduction in Tregs *in vitro*. Results using a pulmonary metastasis model of colon cancer indicated that compared with the control group, ATO group, and CTLs group, metastatic node number decreased significantly (*p*<0.001, *p*<0.001, *p*<0.001, respectively) and survival time was prolonged (*p*<0.001, *p*=0.669, *p*=0.158, respectively) in the ATO plus CTLs group. The number of infiltrated Foxp3+ Tregs decreased in the tumor center, but increased in the peri-tumor tissue. Our results indicate that this approach represents a practical protocol for acquiring autologous CTLs and a feasible strategy that uses a synergistic combination of ATO plus CTLs to treat pulmonary metastases of colon cancer.

## INTRODUCTION

Immunotherapy represents a promising non-toxic anticancer strategy [1, 2], but the various treatment modalities used so far have resulted in only limited and sporadic success [3, 4]. The exact mechanism of successful therapies is unclear, but is often attributed to the reduction of regulatory T cells (Tregs) [5–7]. Increased populations of Tregs have been observed in the blood and tumor tissues of cancer patients [8], where they reduce the number of T helper cells and adversely affect the patient’s prognosis [9]. Current strategies used to deplete Tregs, including low-dose cyclophosphamide and fludarabine [6], irradiation [5], cytotoxic T-lymphocyte-associated protein 4 (CTLA-4) monoclonal antibodies [10], and programmed death-1 (PD-1) blockers [11], only have transient effects.

Arsenic trioxide (ATO) was developed several years ago as treatment for acute lymphoblastic leukemia, and it has subsequently proven to have analogous antitumor effects in many solid tumors, including liver cancer [12] and gastrointestinal tract cancer [13]. Although ATO mono-therapy has limited antitumor effects on solid tumors [14], it could enhance the antitumor activities of 5-fluorouracil, irinotecan, sorafenib [15], PARP-1 inhibitors [16], resveratrol [17], and icariin [18], which the exact mechanism has yet to be explored. A previous study suggested that ATO treatment could deplete Tregs [14], and we also confirmed this mechanism in our previous study [19]. Based on these two studies, we investigated the effects of a combined treatment of ATO and adoptive T cells using a lung metastasis model of mouse colon cancer.

## RESULTS

### Cryopreservation preserved lymphocyte proliferation and killing activity

**Figure 1:**
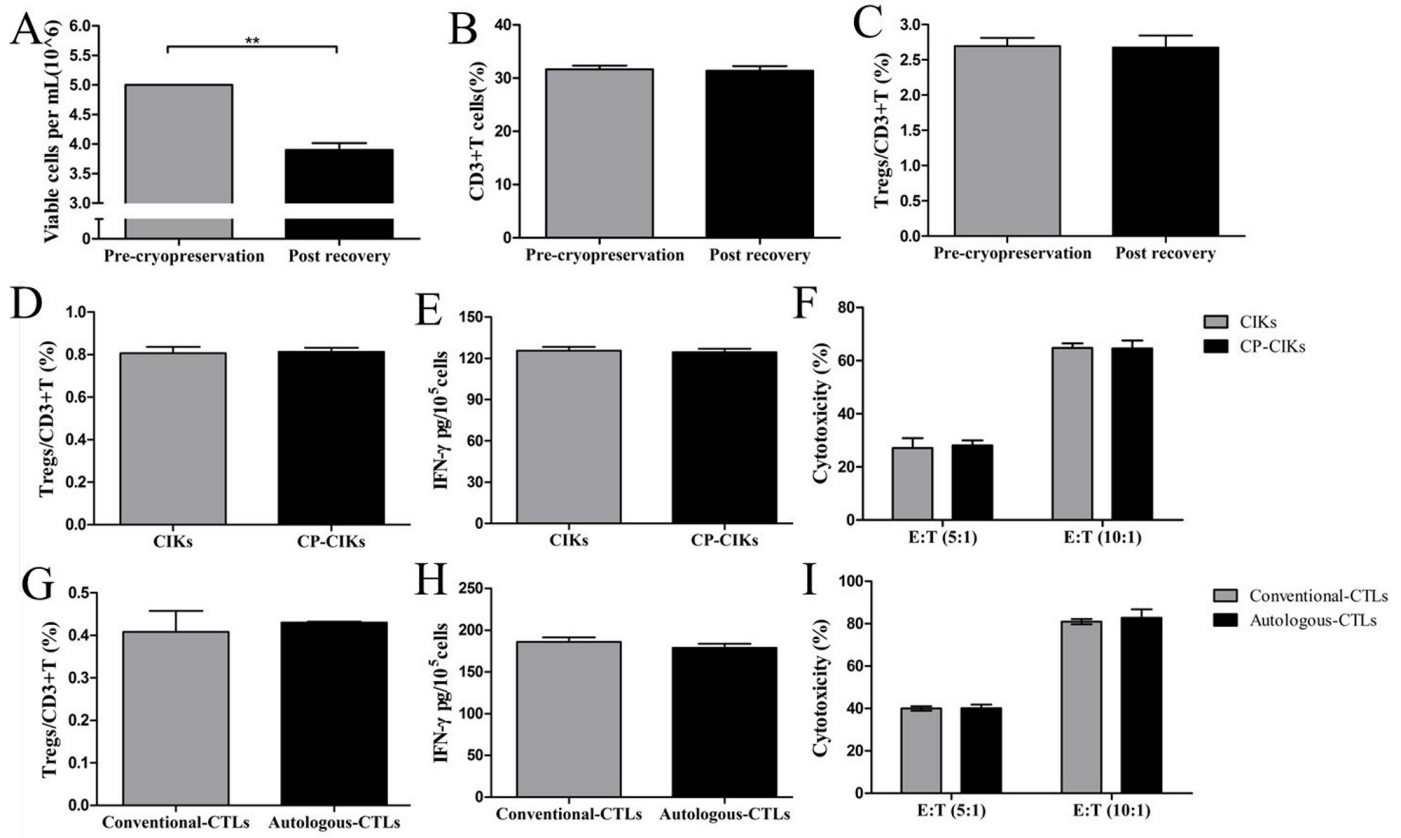
Cryopreservation preserved lymphocyte proliferation and killing activities Naïve spleen T cells were preserved by cryopreservation and then recovered and allowed to proliferate *in vitro* **(A)**. The number of viable lymphocytes after recovery declined significantly compared with the cells pre-cryopreservation **(B, C)**. The percentage of CD3+ T lymphocytes (B) and the ratio of Tregs to CD3+ cells (C) did not change. **(D)** The ratios of Tregs to CD3+ cells were not different between CIK and CP-CIK cells (CP, cryopreserved). **(E)** The levels of supernatant IFN-γ obtained from CIK and CP-CIK cells were equivalent. **(F)** There was no difference in cytotoxicity between CIK and CP-CIK cells. **(G)** The ratio of Tregs to CD3+ cells was not different between conventional CTLs and autologous CTLs. **(H)** The two types of CTLs produced equivalent levels of supernatant IFN-γ. **(I)** There was no difference in *in vitro* cytotoxicity between conventional CTLs and autologous CTLs. CP, Cryopreserved. The *in vitro* experiments were repeated three times. ^*^p < 0.05, ^**^p < 0.01.

We cryopreserved lymphocytes derived from the spleen for 6 days and then recovered at 39°C. After freezing and recovery, the number of viable lymphocytes in the sample, as detected by trypan blue staining, declined significantly (*p* = 0.001, Figure 1A), but the percentage of CD3+ T cells (Figure 1B) and the ratio of Tregs (Figure 1C) did not change. Next, we assessed the capacity of cryopreserved lymphocytes to produce cytokine-induced killer (CIK) cells *in vitro* and found that there were no differences in the ratio of Tregs (Figure 1D), the levels of IFN-γ extracted from their supernatants (Figure 1E), or cytotoxicity (Figure 1F) between CIK and the cryopreserved CIK cells. Finally, we found that there were no obvious differences in the ratio of Tregs (Figure 1G), the levels of IFN-γ (Figure 1H), or cytotoxicity (Figure 1I) between conventional cytotoxic T lymphocytes (CTLs) and autologous CTLs.

### ATO reduced the proportion of Tregs in CTLs cultures and increased CTLs cytotoxicity in a dose-dependent manner

**Figure 2:**
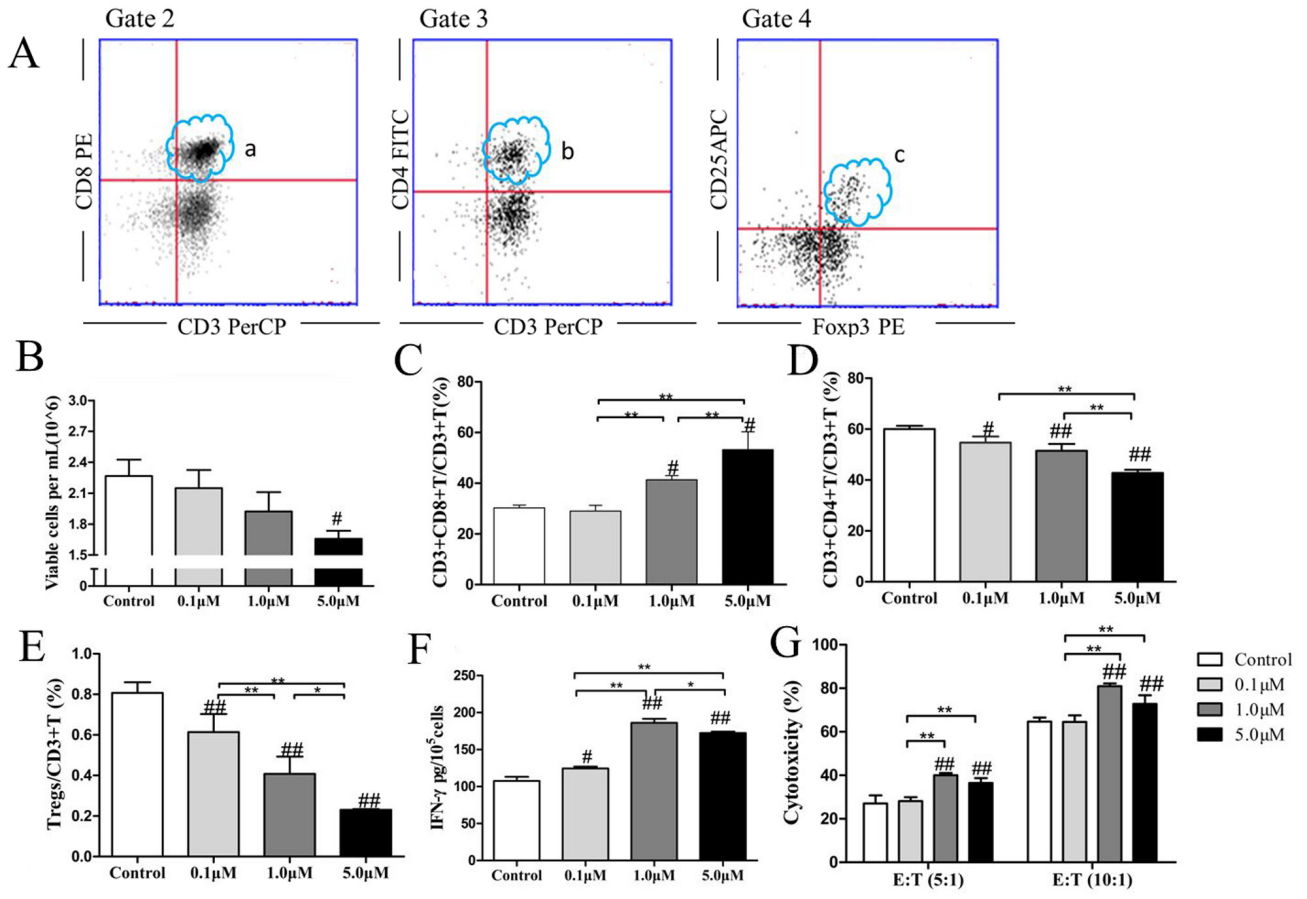
ATO reduced the proportion of Tregs in CTLs cultures and increased CTLs cytotoxicity in a dose-dependent manner The FACS gating strategy was: First, we gated the lymphocyte as Gate 1. Second, we gated CD3+CD8+ cells **(A.a.)** and CD3+CD4+ T cells **(A.b.)** in the right upper quadrants of Gate 2 and Gate 3. Finally, we gated CD3+CD4+ T cells (b) as Gate 4, whose the right upper quadrant was as gated CD25+Foxp3+ Tregs **(A.c.)**. Autologous CTLs were treated with 0.1 μM, 1.0 μM, or 5.0 μM ATO for 48 h. ATO treatment did not significantly alter the total numbers of cells in CTLs cultures **(B)**. However, when we treated CTLs with ATO, the proportion of CD3+CD8+T cells **(C)** increased significantly and the proportions of CD3+CD4+T cells **(D)** and Tregs **(E)** declined significantly in a dose-dependent manner. The levels of supernatant IFN-γ increased significantly following ATO treatment in a dose-dependent manner **(F)**, as did the cytotoxicity of effector CTLs against target CT26 cells at ratios of 5:1 and 10:1 **(G)**. The *in vitro* experiments were repeated three times. ^#^compared with the control group, ^#^*p* < 0.05, ^##^*p* < 0.01; ^*^compared between the experimental groups, ^*^p < 0.05, ^**^p < 0.01.

We gated the lymphocyte as Gate 1 during the first step of the FACS gating strategy. Second, we gated CD3+CD8+ T cells (Figure 2A.a.) and CD3+CD4+ T cells (Figure 2A.b.) in the upper right quadrants of Gate 2 and Gate 3. Finally, we gated CD3+CD4+ T cells as Gate 4, whose upper right quadrant was as gated CD25+Foxp3+ Tregs (Figure 2A.c.). ATO treatment did not alter the total numbers of cells in the CTLs cultures (*p*=0.612, *p*=0.159, *p*=0.025, respectively, Figure 2B). However, when we treated CTLs with ATO, there was a significant increase in the proportions of CD3+CD8+ T cells (*p*=0.691, *p*=0.004, *p*=0.006, respectively, Figure 2C) and a significant decline in the proportions of CD3+CD4+ T cells (*p*=0.012, *p*=0.001, *p*<0.001, respectively Figure 2D) and Tregs (*p*=0.008, *p*<0.001, *p*<0.001, respectively, Figure 2E). These changes were dose-dependent. The levels of supernatant IFN-γ increased significantly following ATO treatment in a dose-dependent manner (*p*=0.021*, p*<0.001 *p*<0.001, respectively, Figure 2F), as did the cytotoxic activity of effector CTLs against target CT26 cells at E:T ratios of 5:1(*p*=0.616, *p*<0.001, *p*=0.002, respectively) and 10:1(*p*=0.930, *p*<0.001, *p*=0.006, respectively, Figure 2G).

### ATO improved CTLs antitumor effects in pulmonary metastases of colon cancer

**Figure 3:**
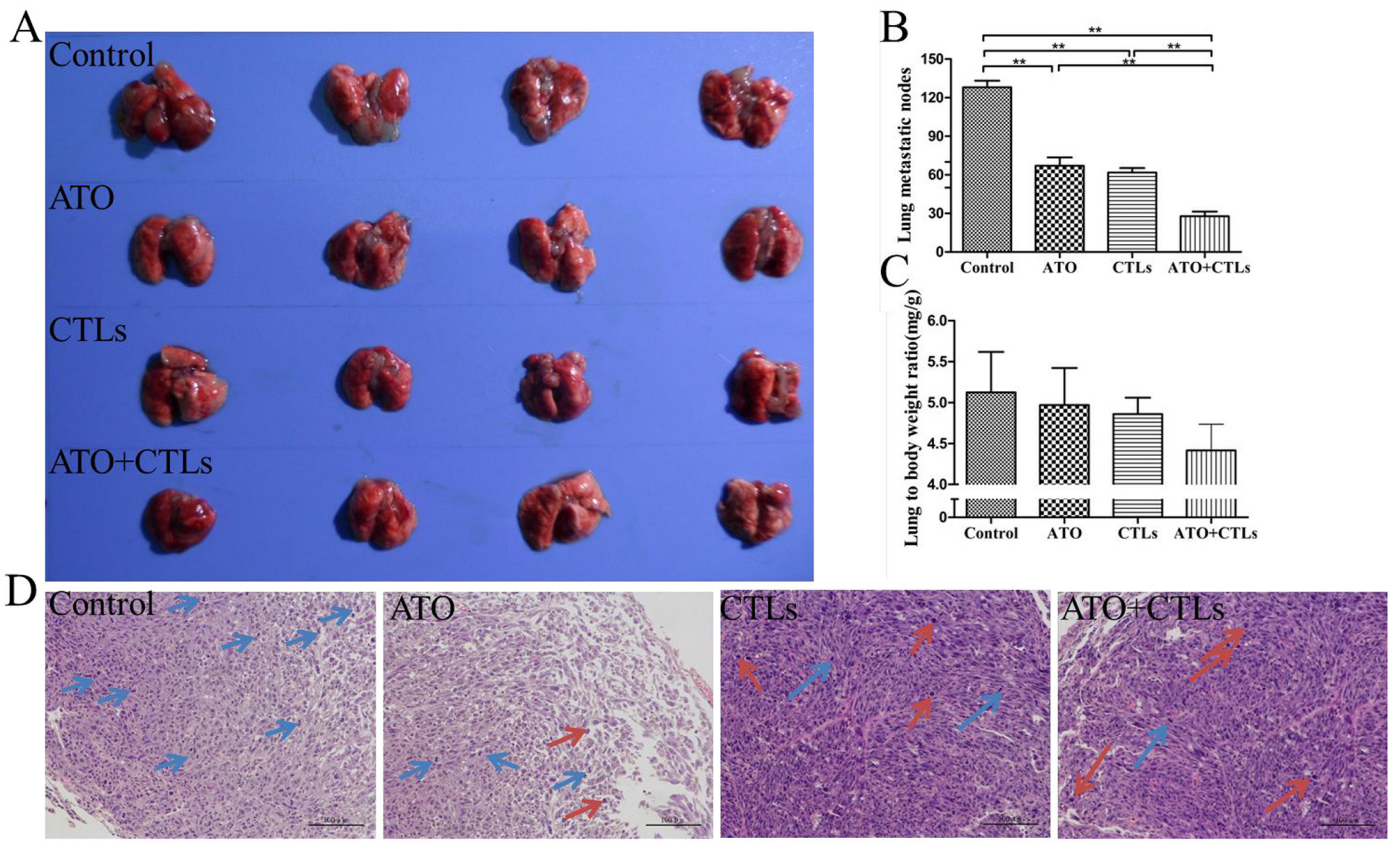
ATO improved CTLs antitumor effects in pulmonary metastases of colon cancer Pulmonary metastatic mice constructed by injecting CT26 colon cancer cells were treated with ATO and autologous CTLs for two weeks. **(A)** Lungs in the treatment groups had smaller nodules. **(B)** The number of metastatic lung nodules decreased significantly in all treatment groups. **(C)** Lung to body weight ratio tended to decrease, without statistically significant differences. **(D)** HE staining revealed that there was more nuclear karyopyknosis in all the treatment groups, compared with the control group (blue arrows indicate nuclear pleomorphism, red arrows indicate nuclear karyopyknosis). ^*^p < 0.05, ^**^p < 0.01, n = 4 per group.

The lung volumes and the number of metastatic lung nodules in the ATO, CTLs, and ATO + CTLs treatment groups were visually smaller compared with that of the control group (Figure 3A). The numbers of metastatic lung nodules decreased significantly in the treatment groups (*p*<0.001, *p*<0.001, *p*<0.001, respectively) compared with that of the control group, the number of nodules in the combined group was the lowest (Figure 3B). On the other hand, the lung to body weight ratio decreased simultaneously, but there were no statistically significant differences among the treatment groups (*p*=0.780, *p*=0.635, *p*=0.216, respectively, Figure 3C). In addition, we found more nuclear karyopyknosis in all the treatment groups by HE staining, compared with the control group (blue arrows indicate nuclear pleomorphism and red arrows indicate nuclear karyopyknosis (Figure 3D).

### ATO and CTLs treatments reduced Tregs infiltration in the tumor center, but increased in the peri-tumor tissue

**Figure 4:**
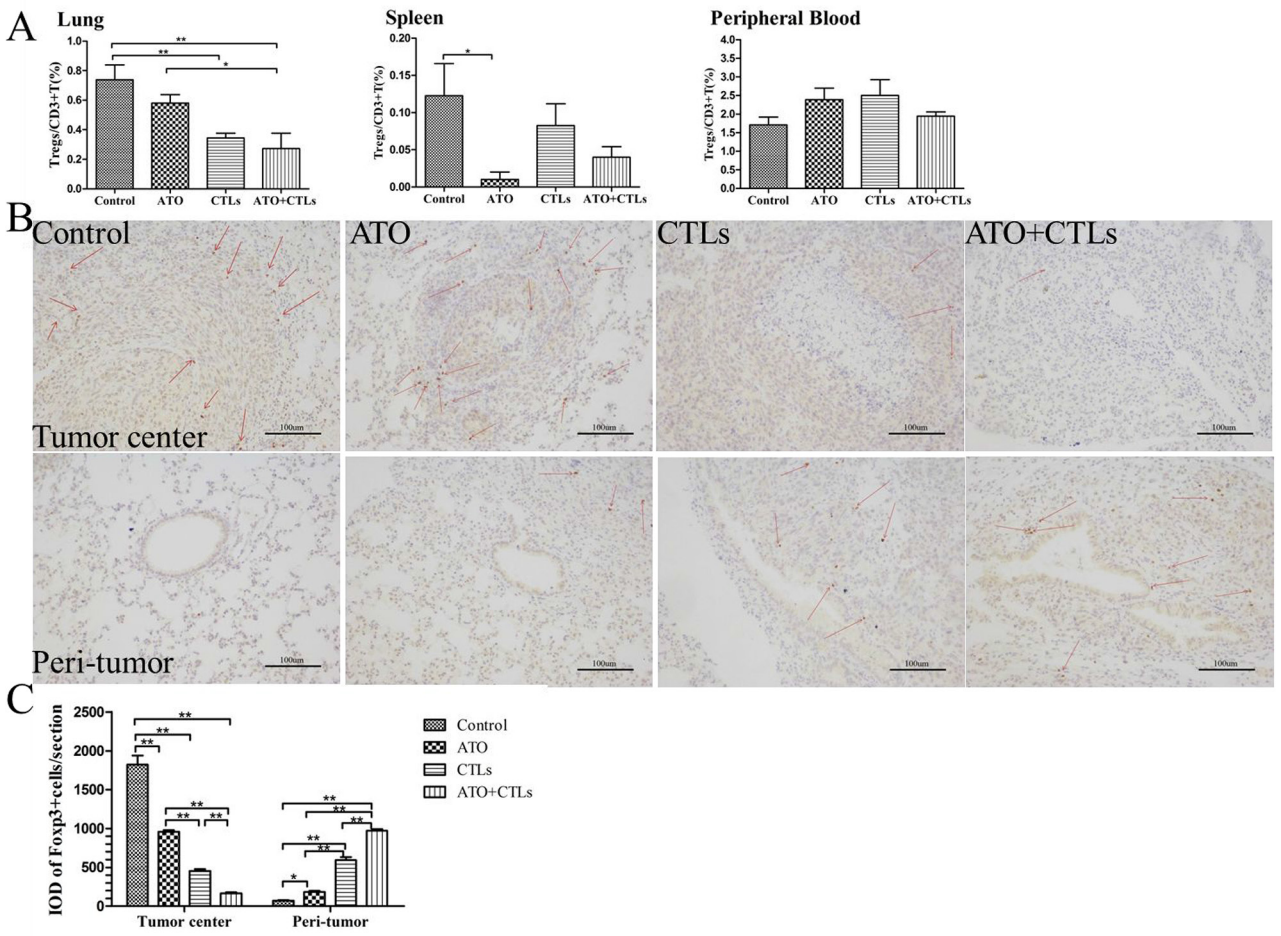
ATO and CTLs treatment reduced Tregs infiltration in the tumor center, but increased in the peri-tumor tissue Tregs were identified using FCM and immunohistochemistry. **(A)** The ratio of Tregs in the treatment groups decreased significantly in the lungs and in the spleen, but not in the blood. **(B)** Immunohistochemical staining of Foxp3 in lung tumor centers and peri-tumors revealed that Foxp3^+^ cell numbers (dark brown) decreased in the tumor center following treatments, but increased in the peri-tumor **(C)**. Quantitative analysis revealed that Foxp3 cell numbers decreased in the tumor center and increased in the peri-tumor significantly. ^*^ *p* < 0.05; ^**^ *p* < 0.01, n = 4 per group.

The ratios of Tregs to CD3+ T cells decreased in the lungs (*p*=0.185, *p*=0.004, *p*=0.001, respectively) in all treatment groups, and in the spleen of the ATO group (*p*=0.013, *p*=0.086, *p*=0.454, respectively); there were no significant changes on the ratio in the blood (*p*=0.121, *p*=0.788, *p*=0.198, respectively, Figure 4A). We used immunohistochemistry to measure the expression of Foxp3 in tumor and peri-tumor sites in the lungs (Figure 4B). We found that the integrated optical density (IOD) of dark brown-stained Foxp3^+^ cells was significantly lower in the tumor centers in the treatment groups compared with the control group (*p*<0.001, *p*<0.001, *p*<0.001, respectively), while it was higher in the bronchus, adjacent to the tumor (*p*<0.001, *p*<0.001, *p*<0.001, respectively, Figure 4C).

### ATO and CTLs treatments increased CD3+T cells infiltrated in the tumor

**Figure 5:**
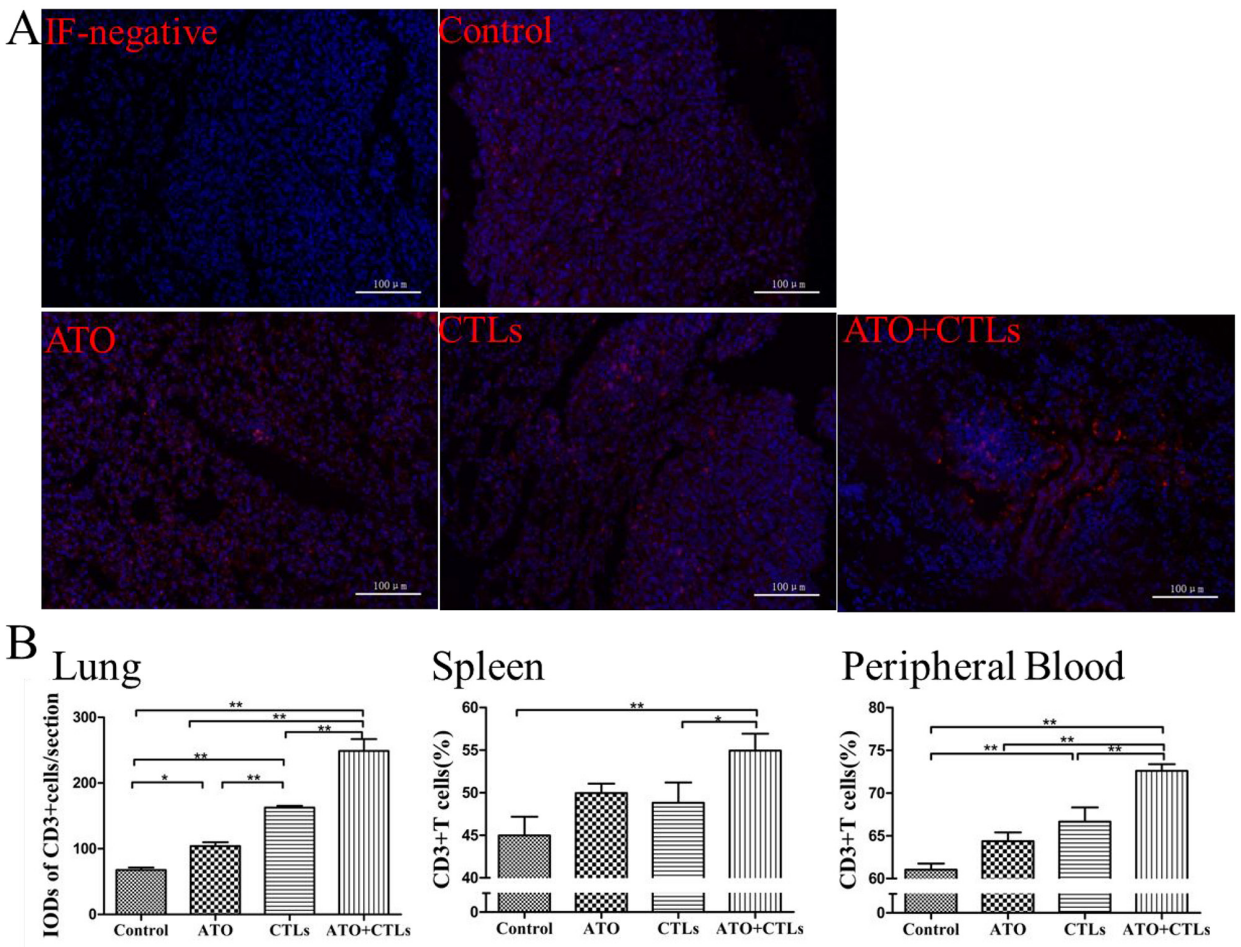
ATO and CTLs treatments increased CD3+T cells infiltrated in the tumor **(A)** CD3+T cells infiltrated in the metastatic lungs were assessed using immunofluorescence. **(B)** In the treatment groups, the integrated optical density of RFP-stained CD3+ cells and the ratios of CD3+T cells in spleen and in peripheral blood were higher, especially in the combination group. ^*^ *p* < 0.05; ^**^ *p* < 0.01, n = 4 per group.

We used immunofluorescence to assess CD3+T cell infiltration in the metastatic lungs (Figure 5A). We found that the values for IOD of RFP-stained CD3+ cells were higher in the treatment groups (*p*=0.020, *p*<0.001, *p*<0.001, respectively, Figure 5B), especially in the combination group. The ratios of CD3+T cells derived from spleen and peripheral blood were measured using flow cytometry (FCM). The results indicated that the ratios of CD3+T cells in the treatment groups increased in spleen (*p*=0.100, *p*=0.193, *p*=0.004, respectively, Figure 5B) and the peripheral blood (*p*=0.055, *p*=0.004, *p*<0.001, respectively, Figure 5B), especially in the combination group.

### Enzymology indicators scarcely changed, and survival rate improved in the treatment groups during ATO treatment

**Figure 6:**
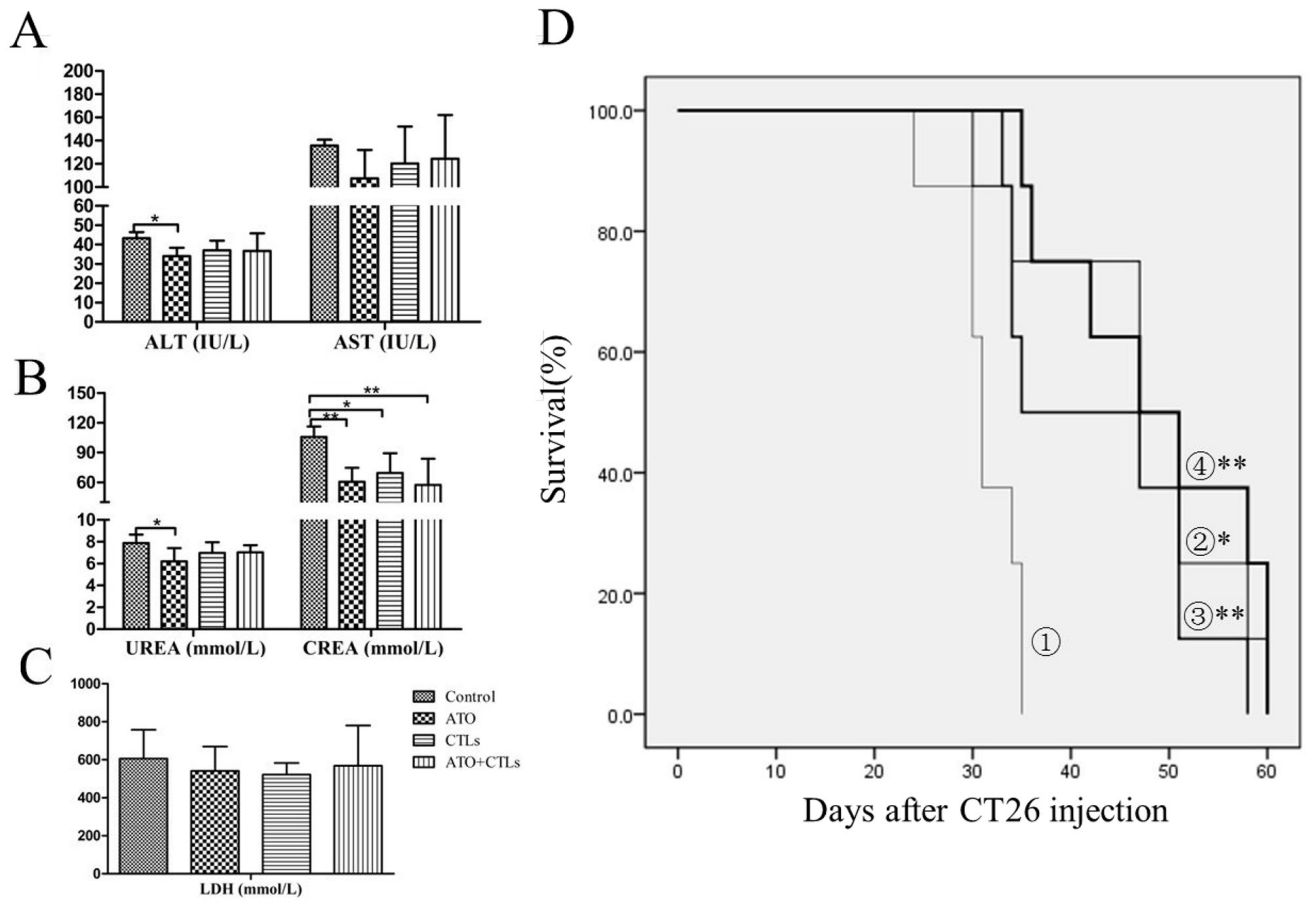
Enzymology indicators scarcely changed and survival rate improved in the treatment groups Liver, kidney, and cardiac enzymology indicators (e.g., ALT, AST, creatinine, urea, and LDH) were measured to evaluate the safety of ATO treatment. **(A)** ALT and AST levels were slightly lower in the treatment groups. **(B)** CREA levels decreased significantly, but urea levels were only lower in the ATO group. **(C)** LDH levels decreased slightly upon treatment, but without a statistically significant difference. **(D)** There were statistically significant improvements in survival rate in the treatment groups (①, control group; ②, ATO group; ③, CTLs group; ④, ATO+CTLs group, respectively), but there were no statistically significant differences among the treatment groups. ^*^p < 0.05, ^**^p < 0.01, n = 6 per group.

The results of the evaluation of the safety of ATO treatment indicated that alanine transaminase (ALT) and aspartate transaminase (AST) levels decreased slightly in the treatment groups, but the differences were not statistically significant (Figure 6A). There were statistically significant decreases in creatinine (CREA) levels (*p*=0.005, *p*=0.018, *p*=0.003, respectively), but the decreases in urea levels were not significant (Figure 6B). The lactate dehydrogenase (LDH) levels dropped slightly, but the changes were not statistically significant (*p*=0.547, *p*=0.437, *p*=0.729, respectively, Figure 6C). Survival rates improved significantly in all three treatment groups, compared with the control group. The control group animals survived 31.25 ± 1.28 days, the ATO group animals survived an average of 47.25 ± 3.73 days (*p =* 0.003), the CTLs group animals survived an average of 42.88 ± 3.52 days (*p=0.012*), and the combined group animals survived an average of 48.63 ± 3.65 days (*p* <0.001). However, there were no statistically significant differences among the treatment groups (*p* > 0.05, Figure 6D).

## DISCUSSION

In this study, we acquired autologous CTLs that were no less cytotoxic than conventional CTLs. We introduced ATO as an adjuvant to deplete Tregs *in vitro*. Lastly, we found that ATO treatment complemented the antitumor effects of CTLs in a model of colon cancer pulmonary metastasis.

We adopted a method of cryopreservation and recovery to preserve naïve T lymphocytes and obtain autologous CTLs. Conventionally, murine CTLs were acquired from mature DCs and naïve T cells, which were derived from different mice. Our cryopreservation method successfully preserved naïve T cells, and once recovered, the cryopreserved T lymphocytes were identical to conventionally-derived co-cultured CTLs both for their ratio of Tregs to CD3+ cells and for their cytotoxicity to CT26 cells *in vitro*.

Increasing frequency of Tregs in tumor diminishes the therapeutic effect of adaptive T cells. We introduced ATO as an adjuvant to deplete Tregs to improve the cytotoxic activity of the adoptive T cells. We found that the cytotoxicity of CTLs against CT26 cells *in vitro* was stronger in the presence of ATO accompanied with decreasing CD4+T cells, Tregs, and increasing CD8+T cells. This result suggested that ATO may have selective effects on CD4+T cells and Tregs. Some studies have revealed that ATO induces apoptosis via oxidative stress in CD4+T cells and Tregs [14, 19, 24, 25], but the exact signaling pathway remains to be determined. The decline in Tregs numbers and the increase in cytotoxicity did not linearly correlate, implying that there might be some unknown pathways that deserve study.

The exact relationship between Treg infiltration and prognosis remains a point of contention. Some researchers argue that low infiltration of Tregs indicates a favorable prognosis [26, 27], some take the opposite position [28, 29], while McCoy et al [30] argue that the relationship does not exist. However, there is some evidence that an increase in Tregs infiltrating in the peri-tumor predicts good prognosis, whereas infiltration in the tumor center predicts bad prognosis [31]. Our findings are consistent with this evidence. In our treatment groups, the frequency of Tregs infiltrating in the tumor center decreased and the frequency in the peri-tumor increased; the same treatments also reduced tumor burden and extended survival time, accompanied with an increase in CD3+T cells. More studies are needed to determine whether the functional significance of this divergence may be an avenue for future investigation.

Finally, we showed that a combination treatment with ATO and CTLs had a synergistic antitumor effect. ATO executes its antitumor effects through multiple mechanisms including direct antitumor effects [32] and indirect immune modulation (e.g., depletion of Tregs) [14, 19]. We introduced ATO as an adjuvant to disrupt immune tolerance for tumors by depleting Tregs. CTLs with a lower proportion of Tregs exhibited enhanced antigen-specificity and killing activity *in vitro*. We successively administered ATO and autologous CTLs to mice. The treatment decreased the number of colon cancer metastatic pulmonary nodules; this result suggested that a synergistic effect was present. The results were not statistically significant, but the improved animal survival rates suggested that the treatment has long-term efficacy to some extent. The tumor persistence-related Tregs recovery and the ATO withdrawal might have contributed to the lack of statistically significant results.

In conclusion, we proposed that our approach represented a practical protocol for acquiring autologous CTLs and a feasible synergistic strategy that combined ATO and CTLs to combat pulmonary metastasis of colon cancer.

## MATERIALS AND METHODS

### Ethics statement

This study was approved by the Ethics Committee of The First Affiliated Hospital of Dalian Medical University (Dalian, Liaoning, China). The experimental mice used in this study were obtained from the Laboratory Animal Center of the Academy of Military Medical Science. All experimental and postoperative animal care procedures were performed according to the protocols approved by the Animal Care and Use Committee of the Chinese PLA General Hospital and the National Institute of Health’s Guidelines for the Care and Use of Laboratory Animals.

### Reagents and cell lines

ATO was purchased from Sigma (St. Louis, MO, USA) and stored at 4°C. Human colon cancer SW-620 cells and mouse colon cancer CT26 cells were obtained from the Institute of General Surgery, Chinese PLA General Hospital (Beijing, China), and cultured in RMPI 1640 medium (Gibco, Grand Island, NY, USA), supplemented with 10% inactivated fetal bovine serum (Gibco, Grand Island, NY, USA), 100 U/mL penicillin, and 100 μg/mL streptomycin at 37°C in a 5% CO_2_ incubator (Thermo Scientific, Waltham, MA, USA).

### Cryopreservation and recovery of lymphocytes

Spleen cells were harvested from Balb/c mice and red blood cells were dissolved with red blood cell lysis buffer (Chinese Academy of Medical Sciences, Beijing, China). After repeated centrifugation and washing, the remaining mononuclear cells were re-suspended at a density of 1 × 10^7^/ml in a cryogenic vial by CELLBANKER 2 (Nippon Zenyaku Kogyo Co., Ltd, Koriyama, Japan). The cells were cryopreserved as follows: 4°C for 3 h, -20°C for 6 h, and -80°C for 2 days stored in liquid nitrogen, and then recovered at 39°C for 5 min, as previously described [20].

### Preparation of mouse CIK cells, CTLs, and *in vitro* study

CIK cells, conventional CTLs and autologous CTLs were prepared in our laboratory as previously reported [19, 21–23]. In an *in vitro* study, CTLs were plated at a density of 2 × 10^6^ /mL in a 100-mm dish and were treated with vehicle control or 0.1 μM, 1 μM, or 5 μM ATO for 48 h. The proportions of CD4^+^ CD25^+^ Foxp3^+^ Tregs and CD3^+^ CD4^+^ T cells in the CTLs were determined using FCM. Briefly, lymphocytes were first incubated with CD3-APC (Miltenti Biotec-REA223, Cologne, Germany), CD4-FITC (Miltenti Biotec-GK1.5, Cologne, Germany), and CD25-APC (Miltenti Biotec-7D4, Cologne, Germany). Then they were subjected to membrane rupture and stained with Foxp3-PE (Miltenti Biotec-3G3, Cologne, Germany) according to the manufacturer’s protocol. All samples were examined by a FACS Calibur instrument (Becton Dickinson, USA) and the data were analyzed with FlowJo7.6.1 software. The level of interferon-gamma (IFN-γ) in the cell culture supernatant was determined using an enzyme-linked immunosorbent assay kit (Sigma-Aldrich, St. Louis, MO, USA). The *in vitro* cytotoxicity of the CTLs (effect cell, E) to the CT26 cells (target cell, T) was assessed using an LDH release assay kit (Sigma-Aldrich, St. Louis, MO, USA) according to the manufacturer’s protocol.

### Animal models and *in vivo* experiments

Six-week-old Balb/c female mice were purchased from the Beijing Experimental Animal Center of the Academy of Military Medical Sciences (Beijing, China). The colon cancer lung metastasis model was established by injecting 1 × 10^5^ mouse CT26 cells in 100 μL phosphate-buffered saline via the tail vein as described previously [19]. At the fourth day, tumor-bearing mice were randomly divided into four treatment groups of 12 mice each. Group 1, 200 μl saline per mouse every day; Group 2, ATO at 3 mg/kg/day; Group 3, CTLs at 1 × 10^7^ per mouse twice a week; and Group 4, ATO at 3 mg/kg/day for the first week, and then CTLs at 1 × 10^7^ per mouse twice during the next week. The treatments were applied for two weeks. At the end of two weeks, six mice from each group were sacrificed to assess the antitumor effect. The remaining mice were observed for 60 days, and data on survival were recorded.

### Processing of lungs

Counting metastatic lung nodules and enrichment of lymphocytes from lung tissue were performed as previously described [19]. Briefly, nodule diameters of less than 0.5 mm, 0.5–1 mm, 1–2 mm, and greater than 2 mm were classified as grade I, II, III, and IV metastasis, respectively. The total numbers of metastases were calculated according to the following formula: total metastasis number = (grade I metastasis number) + (grade II metastasis number × 2) + (grade III metastasis number × 3) + (grade IV metastasis number × 4). The left lung was digested and then the mononuclear cell suspensions were collected using discontinuous density gradient centrifugation with mouse lymphocyte separation medium (MP Biomedicals, Santa Ana, CA, USA). The right lung was processed for HE staining, and Foxp3+and CD3+ staining. Foxp3+ cells were stained brown, and CD3+ cells were conjugated to a red fluorescence protein. Image-Pro Plus 6.0 software was used to convert fluorescent images to black and white images, which were used for IOD assessment.

### Statistical analysis

SPSS 21.0 statistical software was used for statistical analysis of the relevant data. Data are expressed as the mean ± standard deviation. Differences between two groups were compared using T-tests. Differences among several groups were analyzed by one-way analysis of variance. *P* < 0.05 was considered statistically significant. The survival rate was analyzed using the Kaplan-Meier method.

## Abbreviations

CTLs: cytotoxic T lymphocytes
Tregs: regulatory T cells
ATO: arsenic trioxide
CIK: cytokine-induced killer
CP: cryopreserved
IOD: integrated optical density
ALT: alanine transaminase
AST: transaminaseaspartate transaminase
CREA: creatinine
LDH: lactate dehydrogenase
